# Designing a minimum dataset for autism spectrum disorder registry in Iran

**DOI:** 10.1097/MS9.0000000000000496

**Published:** 2023-04-18

**Authors:** Monir Shayestehfar, Pardis Jahandideh, Rabeeh Hariri, Malihe Shayestehfar, Amirhossein Memari

**Affiliations:** Sports Medicine Research Center, Neuroscience Institute, Tehran University of Medical Sciences, Tehran, Iran

**Keywords:** Autism spectrum disorder, MDS, Minimum dataset, Registry

## Abstract

The reported prevalence of autism spectrum disorders (ASDs) has been showing a marked increase over the past 20 years. Therefore, a uniform data gathering system for ASD registration could prominently enhance plans for managing ASD worldwide. So, in the current investigation the authors aimed to design and validate the Persian version of a minimum dataset (MDS) for being administered in the national ASD registries.

**Methods:**

The current study is a mixed-method study with both quantitative and qualitative methods providing and validating a form of MDS in four phases according to Delphi method. The proposed MDS consisted of 11 categories containing coding responses. Content validity (CV) was evaluated based on 20 expert’s suggestions and opinions. Item-CV Index (I-CVI) and Scale-CVI were administered to evaluate and validate the items and questions in the proposed MDS.

**Results:**

Twenty researchers from different disciplines scored each question and item. By taking into account the scores, the validity appraisal was provided for each item by computing the I-CVI value. Results showed that 41 out of 76 items had the value I-CVI less than 0.78 and were kept as relevant; 35 items were eliminated due to a value below 0.70. The Scale-CVI /Ave of the relevance for the entire form was 0.9396.

**Conclusion:**

The Persian version of MDS for ASD registry was found to be valid. Such MDS can be utilitarian for health cares and policymaking purposes by gathering and updating standard data for developing local and national registries.

## Introduction

HIGHLIGHTS

- We have introduced a valid minimum dataset for recording the basic information of individuals with autism spectrum disorder.
- In such minimum dataset, we have provided a range of basic, primary, and necessary information that could be collected from simple demographic information to more detailed medical and diagnostic data on each patient with autism spectrum disorder.
- This study may facilitate designing national registries, and improve the quality of healthcare services by quick accessing to data, and easily retrieving to the unit information about the individual.

Autism spectrum disorder (ASD) is a neurodevelopmental disorder characterized by impairments in social communication and interaction and restricted and repetitive patterns of behaviour, interests, and activities. The reported prevalence of ASDs has been showing a marked increase over the past 20 years for not completely clear causes^1^. Complex genetic factors, as well as a variety of non-genetic and environmental factors, have been reported in aetiological research on ASD^2^–^4^. The aetiological complexity and vast heterogeneity of ASDs have prompted the establishment of national and multinational data gathering systems or registries to facilitate data sharing and collaborative analyses of pooled data^5^. Registries can help to better understand the condition by collecting consistent data on different aspects of that specific disease^1^. In case of ASD, there are several registry systems such as the International Collaboration for Autism Registry Epidemiology (iCARE), Autism Treatment Network (ATN) registry, and the New Jersey autism registry. For example, iCARE has been aiming to encourage and promote research projects in geographical and temporal heterogeneity of ASD, aetiology, phenotype, family and life course patterns^6^. In addition, this international ASD registry devises solutions for challenges in multinational collaboration in regard to data access security, confidentiality and management^6^.

In a specific registry, a range of information could be collected from simple demographic information such as number, gender, date of birth, and primary language to more detailed medical and diagnostic data on each patient. Administrating a minimum dataset (MDS) for recording the individuals with a specific disease and the follow-up data can improve the quality of healthcare services and decrease the costs^7^. A MDS is a standardized core set of information required for guiding data collection in a registry. MDS appears critical for creating a database by facilitating primary or minimum collection and processing of the data. Furthermore, it can be used for standardizing and assimilating broad views of different healthcare settings on healthcare services^7^. In other words, MDS provides an opportunity for strengthening effective communication between researchers and research outputs, improving programs, policies, and strategies^8^.

The MDS will benefit the registry system by quick accessing to data, enabling evaluation and identification of inequalities in service delivery and outcomes, preventing duplicate records, and easily retrieving and access to the unit information about the individual with a specific disease. In addition, MDS improves the quality of care by continuous monitoring of the disease process, and effective allocation of health resources, which saves time^9^. Determining MDS plays an essential role in the registration process for a particular disorder. There are studies introducing and validating MDS for several disorders such as Multiple Sclerosis (MS)^10^, breast cancer^8^, stroke^11^, and spinal cord injury^12^. However, in spite of the existence of several national and international registries for ASD, as far as we know, there is still no study introducing and providing the MDS for being administered in databases and registry systems.

Thereby, it could be a significant missing link for healthcare professionals, parents, and children in order to utilize treatments, programs, and follow-ups that affect prevalence rates, symptoms, and family’s mental health. This article claims to be in an effort to design a MDS for autism registry in Iran by exploring compiled data.

## Methods

The current investigation is a mixed-method study with both quantitative and qualitative methods. The population of this study included experts in the fields of neuroscience, neurology, paediatrics, epidemiology, nursing, and clinical psychology. Since in the most related Delphi studies, the number of experts in the committee was 15–20, twenty experts were selected in this study based on purposive/non-random sampling. The inclusion criteria were as follows: (1) Having at least three years of practical experience and sufficient expertise with children with ASD, (2) being familiar with the ASD literature, and (3) having the age range between 35 and 55 years. In addition, the exclusion criteria were not having the preference to continue the project at any stage of the study. This study has been conducted with the aim of validation of MDS for national registration of ASD according to the following steps:

### Phase 1

A systematic review was conducted to determine MDSs for the existed ASD registries in order to create and retrieve the relevant minimum data elements. Authentic databases were searched accurately, including PubMed, Scopus, Web of Science, and the Google Scholar search engines. The key terms used in the search strategy were as followings: MDS OR “minimum data” OR “minimum data set” AND autism OR “autism registration” OR “autism registry” OR “ASD registration” OR “ASD registry.” The inclusion criteria for the selected studies were English published studies between 1990 and 2022.

### Phase 2

A list of data elements for MDS was compiled and categorized based on the information provided in the first phase. Then, the expert committee reviewed the collected data, and the required modifications (such as the comprehensibility of the items) were applied to the provided data lists. Finally, the essential elements of MDS required for a national ASD registry was proposed based on the professional opinions of the expert panel.

### Phase 3

In order to assess the validity and reliability of the proposed MDS, we administered both quantitative and qualitative content validity based on the opinions of the expert panel. The expert committee members were asked their views on the qualitative content review. They were requested to submit their corrective ideas and suggestions in a text after carefully studying the proposed MDS using Delphi technique in two rounds. In addition, a team of experts examined each item in the questionnaire separately, using the Content Validity Index (CVI) in order to assess quantitative content validity. Based on the Delphi technique, a two-part checklist was designed in this regard. The first part included a question about preserving or removing any piece of a data element from the MDS. In the second part, the evaluation and ranking of the MDS were assessed based on the importance of each data component using a 4-point Likert (from “*not-important*”: 1, to “*Very Important*”: 4). Furthermore, the expert panel members were asked to add any additional items or suggestions beyond the questionnaire. Data elements with a score of higher than three were maintained in the proposed MDS, and objects with a score of lower than two were removed. In addition, data components with a score between 2 and 3 and extra elements proposed by the expert panel were entered the second round of the Delphi technique. Finally, the proposed MDS included the following dimensions:

Demographic information, Family history, Environmental factors, Diagnostic Information, Therapeutic strategies/drugs, Disease progression, and Visits/follow-ups.

Considering the above description, the proposed model was acculturated according to the country’s requirements. The final version of the submitted form consisted of 50 items containing coding responses. The last items could be classified as follows: identification, familial history, education, birth and infancy information, diagnosis, developmental history, child’s medical history and medication, care system providers, and research participation.

### Phase 4

In the last phase, in order to continue the validation procedure and confirm the feasibility of MDS application in the field, the final draft of MDS approved by the Experts Committee was examined in a convenient sample of ASD aged ranged 6–17 years. In this regard, 50 healthcare providers (i.e. physicians and nurses) were asked to complete the MDS items for a convenient sample of ASD. They were also asked to provide feedback on interpretability, clarity or possible ambiguity of the phrases or statements.

### Statistics

Item-CVI (I-CVI) which is indicator of the validity of question, was measured for each item separately. The critical value for I-CVI is considered 0.78^13^. Moreover, the content validity of the total form was measured by Scale-CVI/Average (S-CVI/Ave) which addresses the relevance, clarity, and simplicity of the dataset^14^. The critical value for S-CVI/Ave is considered 0.9^14^.

## Results

### Descriptive

The expert committee consisted of 20 researchers, including eight men and 12 women, with a mean age of 39.5±4.7, scored the questions. The members of committee were experts in different fields including neuroscience, neurology, paediatrics, epidemiology, nursing, and clinical psychology. Beside checking for validity of the items, the expert committee also checked the items semantically. In case of the need for any modification in the meaning of the items, the expert committee members discussed and change the item in a way to be more comprehensible.

### Validity

By taking into account the scores, the validity appraisal was provided for each item by computing the I-CVI value, which 41 out of 76 items had the value I-CVI less than 0.78 and were kept as relevant; 35 items were eliminated due to a value below 0.70. Table 1 shows the eliminated items and their I-CVI scores. The examples of eliminated items are: parents’ education, caregivers’ occupation, seizure/icterus at birth, required PCR at birth, and mother’s infertility history.

**Table 1:** Content validity indices of the remaining questions in MDS for ASD registry

	Categories and items	ICV score
Identification		
1	Full name	0.95
2	Sex	1.00
3	Birth date	1.00
4	National ID number	1.00
5	Address	0.80
6	Phone	0.90
7	Dominant hand	0.85
8	Education	1.00
9	Insurance/type	0.95
Familial history		
10	Parents/caregivers medical history	0.80
11	Autism family history	1.00
Education		
12	Type of school/education	1.00
Birth and infancy information		
13	Pregnancy duration	0.95
14	Type of delivery	0.95
15	Birth weight	0.80
16	Birth order	0.90
17	Birth defects or problems	0.90
18	Vaccination	0.85
19	Breastfeeding	0.90
Diagnosis		
20	What was the diagnosis?	0.95
21	Who did diagnose?	0.90
22	Age of the diagnosis	0.95
23	Where was this diagnosis provided?	0.85
24	Diagnosis instruments	0.95
25	At what age did your child first show a developmental problem?	1.00
26	The main concern of caregiver about the child development (before diagnosis)	1.00
27	IQ test	0.95
28	ADOS test	0.85
29	ADI-R test	1.00
30	MRI, CT-scan, EEG test	0.80
Developmental history		
31	Age of the first walk	1.00
32	Age of the first talk	1.00
33	Which type of skills was most affected?	0.90
Child’s medical history and medication		
33	Comorbidity diseases	1.00
34	Medications prescribed due to the diagnosis	1.00
35	Other treatments	0.95
Quality of care		
36	Associations membership member of an association?	1.00
37	Examination schedule	0.90
38	Quality of care by healthcare staff	0.85
39	Quality of family training about autism care by healthcare staff	0.90
40	Sources of information about autism	0.90
Research participation		
41	History of participation in autism research	1.00
42	S-CVI/Ave	0.93

ADOS, Autism Diagnostic Observation Schedule; ADI-R, Autism Diagnostic Interview-Revised; ASD, Autism spectrum disorder; CT, computed tomography; EEG, electroencephalogram; IQ, Intelligent Quotient; MDS, minimum dataset; S-CVI, Scale-Content validity index.

Table 1 shows the remaining items in the questionnaire and their I-CVI scores. The S-CVI/Ave of the relevance for the entire form was 0.9396 (Table 1).

In addition, in a pilot investigation 49 out of 50 healthcare providers of children with ASD could complete the form without any serious misunderstanding of the questions indicating the simplicity and clarity of the items in the final form.

## Discussion

According to the latest estimates of centres for disease control and prevention in 2021, ~1 in 44 children in USA is diagnosed with an ASDs. Although ASD can be diagnosed reliably as early as age 2, most children are still being diagnosed after age 5 or even later particularly in developing countries. Early diagnosis and preventive proceedings can improve the results and reduce the incidence of new cases^15^. Besides, the increasing and variety of daily living problems for ASD individuals and their families are one of the most significant challenges for healthcare systems in societies.

The lack of tools for standard data collection and the absence of a comprehensive national dataset for ASD may exacerbate the confusion and chaos of ASD management and variable content of ASD information in societies particularly developing countries. In other words, valid tools for standardization of data can improve prominently the regional, national, and international challenges related to the policymaking and service providing of a specific disease. To achieve this goal, setting up a standard system for recording data on ASD, accelerate the diagnosis by compiled information. ASD registry in Iran is an information system that includes collecting, classifying, coding, and sorting data related to the disorder reported from birth to age 21 by healthcare centres, schools and welfare centres. In this regard, development of a standard MDS for a particular disorder such as ASD is required for a comprehensive collection of data in different societies and countries. It is worth mentioning that the value of a MDS is not only for registration systems, but also for forming primary and basic electronic files in the clinics and hospitals’ databases.

In the current study, the diagnosis items were notably selected by considering the American Psychiatric Association’s Diagnostic and Statistical Manual, Fifth Edition (DSM-V), which provides standardized criteria to help diagnose ASD. We use three methods of developing the MDS: individual sessions with experts, Delphi technique, and specialized group discussions. According to the study results, 76 questions were evaluated by experts. Fifty-three items attained sufficient content validity; however, 23 items were eliminated due to not gaining terms of validity. Finally, the proposed MDS retained high validity scores. As a result, the proposed MDS included the demographic characteristics of the individuals with ASD and their parents, medical/psychiatric history of the individuals with ASD and their parents, diagnosis information, developmental history, pregnancy and birth information, medications, and treatments. The full information about pregnancy and pregnancy-related problems were not included in the final MDS. Furthermore, education items, such as having an assistant in a school, and permission to attend the regular school, were not included in the MDS for ASD.

This study attempted to design a standard and validated MDS to further expand ASD registry and improve national collection of ASD data in Iran. There are numerous ASD registry systems throughout the world and undoubtedly each registry system has its own particular required minimum data. However, to our knowledge there is no study introducing and validating such MDS specific for ASD registry to the scientific literature. In this vein, we proposed and designed a MDS and assessed its validity in the second step. A valid and reliable tool is of paramount importance for collecting standard data and performing research or clinical projects and therefore should be taken into consideration in the literature and disease registration^16^. Determining healthcare equality across different regions of the country, running clinical, aetiological, and epidemiological researches, developing an efficient follow-up system, as well as assessing the quality and cost-effectiveness of healthcare services are of major objectives of ASD registration in Iran which are similar to those registry systems in the developed countries.

There might be a few differences between the provided MDS in the present study and the unknown MDS in other ASD registries in terms of the details of data collection, documentation, and even scope of registrations. However, improving current knowledge and understanding of ASD is an important common point in all ASD registration systems.

### Limitation and future direction

In spite of the several strengths of our study such as administrating a multicentre cooperation of experts and conduction of a pilot study, lack of longitudinal reliability assessment was one of the limitations of the current study. We recommend future studies to further examine MDS for ASD registrations and also try to develop the multicentric networks by connecting the datasets using the current proposed MDS.

## Conclusion

A designed and validated MDS for ASD registry was proposed in the current study. The list of items was assessed and validated by an expert committee and modified according to the analyzing scores. The MDS can be utilized for healthcare and policymaking purposes by gathering and updating standard data for developing local and national registries by means of a set of standardized validated minimum data.

## Ethical approval

This study was approved by ethics committee of our university of medical sciences (we are supposed to state unanimously).

## Consent

The parents of all participants signed the informed consent for participating in this study.

## Source of funding

NA.

## Conflicts of interest disclosure

There is no conflict of interest to declare.

## Provenance and peer review

Not commissioned, externally peer-reviewed.
